# Intussusception causing postoperative intestinal obstruction following free jejunum transfer in adults: two case reports and review of the literature

**DOI:** 10.1186/s40792-015-0028-2

**Published:** 2015-03-11

**Authors:** Akira Matsumoto, Masayuki Watanabe, Hironobu Shigaki, Yasuhiro Okumura, Koujiro Nishida, Shinji Mine, Kazuhiko Yamada, Katsuhiko Yanaga, Takeshi Sano

**Affiliations:** Department of Gastroenterological Surgery, Cancer Institute Hospital of Japanese Foundation for Cancer Research, 3-8-31 Ariake, Koto-ku, Tokyo 135-8550 Japan; Department of Surgery, Jikei University School of Medicine, 3-25-8 Nishi-shinbashi, Minato-ku, Tokyo Japan

**Keywords:** Intussusception, Free jejunum transfer, Bowel obstruction, Complication

## Abstract

Intussusception is a rare cause of postoperative intestinal obstruction in adults. We experienced two cases of bowel obstruction due to the jejuno-jejunal intussusception after harvest of a free jejunum graft for reconstruction after cervical esophagectomy. Bowel obstruction occurred early in the postoperative course, and reoperations were needed in both cases. In both case, the anastomotic site was resected and re-anastomosed in a side-to-side fashion. Recurrence of intussusception has not been observed. In the literature, such a complication has been documented in two case series and a case report. The reported incidence of postoperative intussusception of the case series was 2.8% and 7.4%, respectively. The jejuno-jejunal anastomoses were performed with end-to-end fashion by two layered hand-sewn suture (Albert-Lembert method) in all cases reported. In order to prevent the occurrence of postoperative intussusception, we recommend to harvest a free jejunal graft as far from the Treitz ligament as possible and to avoid reconstruction by an Albert-Lembert end-to-end anastomosis.

## Background

Intestinal intussusception is a relatively common abdominal emergency in children, whereas incidence of intussusception in adults is rare and represents less than 5% of all cases [1]. Almost 90% of adult intussusceptions are secondary to pathologic conditions such as polyps and neoplasms that serve as a lead point [2-4]. Intussusception after abdominal surgery in adults is a relatively rare disorder and with a few reports on postoperative intussusception after gastrectomy [5-8].

Free jejunum transfer (FJT) is the most common method of esophagopharyngeal reconstruction after pharyngolaryngectomy or cervical esophagectomy because of its low incidence of complications and sufficient quality of oral intake [9-12]. We herein report two cases of bowel obstruction due to jejuno-jejunal intussusception which occurred after harvest of a free jejunum graft. In addition, we reviewed the literature on this rare complication after FJT.

## Case presentation

We experienced two cases of postoperative bowel obstruction due to jejuno-jejunal intussusception at the site of anastomosis after harvest of a free jejunal graft. A literature search was conducted with PubMed database and Ichushi database of Japan Medical Abstracts Society using the keywords adult intussusception ‘OR’ bowel obstruction ‘OR’ complication ‘AND’ free jejunal transfer. We found two case series and one case report that documented such a rare complication after FJT [13-16]. We retrieved data on the harvested grafts, anastomotic methods during the first surgery, timing of reoperation, and procedures of the second surgery.

### Case 1

A 75-year-old Japanese man underwent cervical esophagectomy for recurrent esophageal cancer at the cervical esophagogastric anastomosis. The esophageal reconstruction was performed with FJT. We harvested a free jejunal graft with the third jejunal artery as the vascular pedicle, and jejuno-jejunal anastomosis was performed in a two-layered end-to-end fashion with Albert-Lembert method. On postoperative day (POD) 21, he vomited without abdominal pain, and complete obstruction of the upper jejunum was suspected by plain abdominal X-ray. Computed tomography (CT) demonstrated a bowel-within-bowel configuration in the upper small intestine. Although we suspected that the cause of small bowel obstruction as intussusception, there were no signs of strangulation. Therefore, we started conservative treatment with bowel decompression using a long intestinal tube. Although bowel obstruction improved within a few days, it recurred after resumption of oral intake. Then, we performed re-operation on POD 42. In the abdominal cavity, there was a jejuno-jejunal intussusception with the end-to-end jejunal anastomosis as a lead point. The anastomotic site was resected, and bowel reconstruction was performed by functional end-to-end anastomosis. He has never experienced bowel obstruction for 11 months after the second surgery when he died of recurrent esophageal cancer.

### Case 2

**Figure 1:**
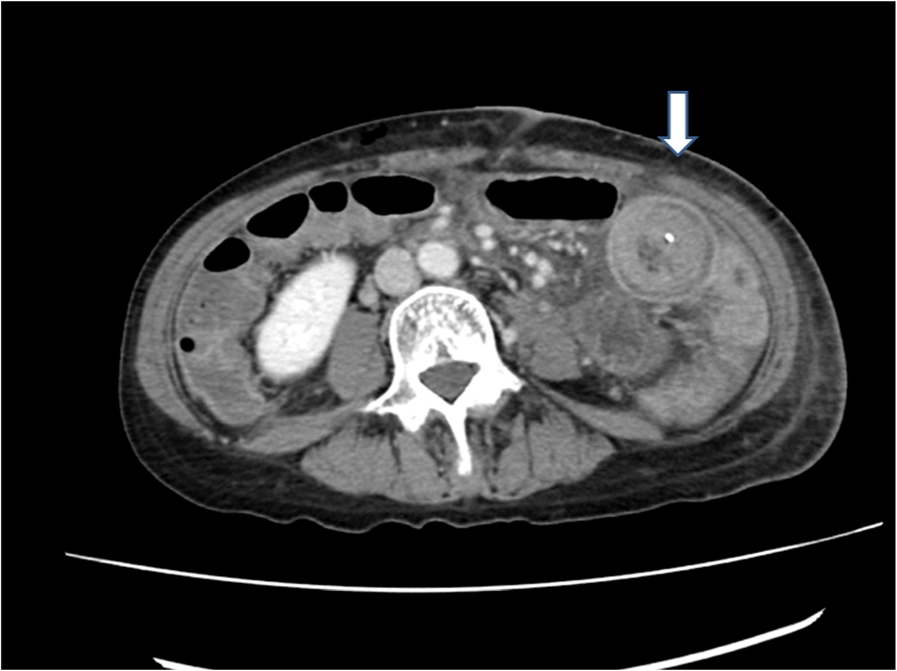
Abdominal computed tomography. A multiplex ring-formed mass connected with the small intestine.

**Figure 2:**
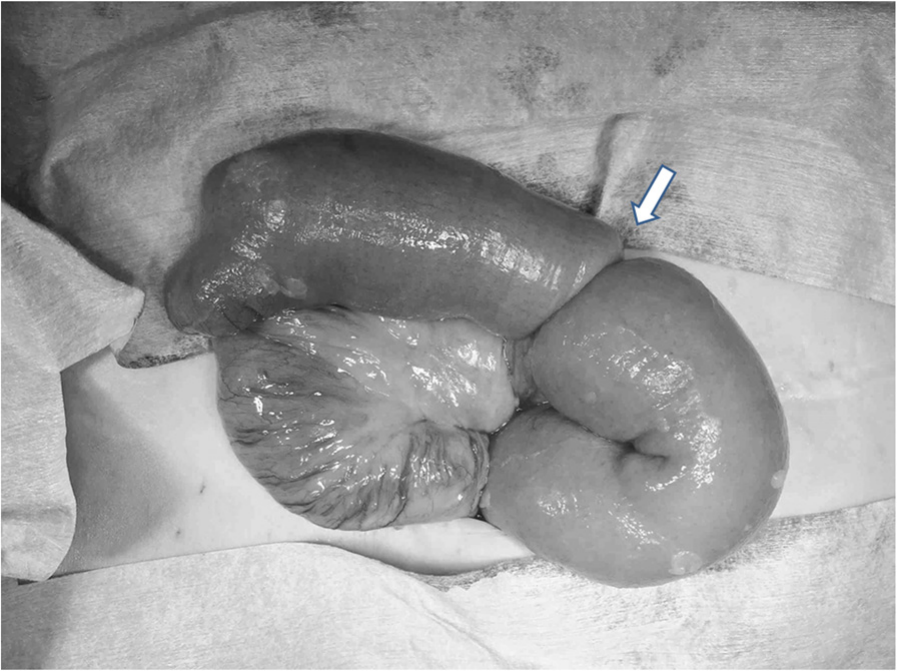
Intraoperative finding. Intussusception was caused by intestinal anastomosis.

A 67-year-old women underwent cervical esophagectomy for second primary cervical esophageal cancer. Esophageal reconstruction was performed by FJT, using a jejunal graft with the third jejunal artery as the vascular pedicle, and the jejuno-jejunal anastomosis was performed in an end-to-end fashion by the Albert-Lembert method. On POD 3, she vomited and bowel obstruction was suspected. An abdominal CT demonstrated a bowel-with-bowel configuration, suggesting intussusception (Figure 1). We diagnosed the cause of bowel obstruction as intussusception over the jejuno-jejunal anastomosis, and reoperation was performed. In the abdominal cavity, intussusception due to the anastomosis as a lead point was identified (Figure 2). The anastomotic site was resected, and the jejunum was reanastomosed in a side-to-side manner. She has been alive without symptom of bowel obstruction for 8 months after the reoperation.

### Characteristics of patients who experienced intussusception after FJT

A review of the literature revealed eight cases of intussusception occurring after FJT, including the current cases. In all patients, the lead point of intussusception was the jejuno-jejunal anastomosis. All patients underwent reoperation, and almost all of the patients required bowel resection. Operative procedures for harvesting the free jejunal grafts were very similar among all cases: The free jejunal grafts were harvested at the area of second or third jejunal arteries, and jejuno-jejunostomies were performed in an end-to-end fashion by the Albert-Lembert method. Side-to-side anastomoses or end-to-end anastomoses in a layer-to-layer fashion were performed at the second surgery. No recurrent intussusception has been reported in these cases (Table 1).

**Table 1:** **Characteristics of patients who developed intussusception after free-jejunal transfer**

**Reference**	**Year**	**Age (years)**	**Sex**	**Jejunal artery used for free jejunal graft**	**Method of anastomosis at first surgery**	**Interval: primary operation to reoperation (days)**	**Bowel resection**	**Method of anastomosis at second surgery**
Flynn et al. [16]	1989	NS	NS	NS	NS	NS	Yes	NS
Omura et al. [13] Urayama et al. [15]	1993 1997	76	F	Second	End-to-end A-L	20	Yes	Side-to-side A-L
		64	M	Third	End-to-end A-L	28	No	None
		38	M	Second	End-to-end A-L	90	Yes	End-to-end layer-to-layer
		53	F	Third	End-to-end A-L	19	Yes	End-to-end layer-to-layer
Kawasaki et al. [14]	2007	75	F	Third	End-to-end A-L	48	Yes	Functional end-to-end
Current report	2014	75	M	Third	End-to-end A-L	42	Yes	Functional end-to-end
		67	F	Third	End-to-end A-L	3	Yes	Fide-to-side A-L

NS, not stated; M, male; F, female; A-L, Albert-Lembert suture.

## Discussion

Intussusception is a rare cause of postoperative intestinal obstruction in adults. It occurs in only 0.07% to 2.1% of individuals who undergo gastrectomy, although 87.7% of intussusceptions following abdominal surgery occur after gastrectomy [7,17]. In the literature, intussusception after FJT was reported in two case series and a case report [13-16]. In these articles, we found only six cases of intussusception after FJT. The reported incidence of postoperative intussusception of the case series was 2.8% and 7.4%, respectively. In our institution, we performed FJT reconstruction for 218 patients from January 2011 to December 2014, and only the reported two cases (0.9%) developed intussusception.

Although the exact mechanism of intussusception remains unclear, some of the clinical features are common and the others are different between intussusceptions after FJT and those after gastrectomy. One of the common features was that invagination occurred at the anastomotic site in the proximal jejunum. Many cases of post-gastrectomy intussusception were associated with Billroth II or Roux-en-Y reconstructions, while those associated with Billroth I reconstructions were rare [1,18]. Intussusceptions after gastrectomy frequently occurred in the region of the anastomotic entrance, including the Braun’s anastomosis [19]. High motility and relatively large enteric diameter of the proximal small intestine may influence the occurrence of invagination. On the other hand, intussusceptions after gastrectomy often appeared as retrograde invagination, whereas normograde ones were observed in all cases occurred after FJT. Although the lapse of time between gastrectomy and the occurrence of intussusception has been reported as from 2 days to 30 years, post FJT intussusceptions occurred early in the postoperative period. The differences in clinical features may suggest the difference in mechanism of occurrence between both situations, and transient edema at the anastomotic site may be a main cause of intussusception after FJT.

The Albert-Lembert method, which is one of the most popular technique for bowel anastomosis, is double-layered hand-sewn anastomosis consisted of full-layer suture and seromuscular suture. Intussusception after FJT has occurred after Albert-Lembert method in all cases. Although the Albert-Lembert method provides sufficient tensile strength and hemostasis, the suture line tends to become thick and transient edema after anastomosis may cause temporal anastomotic stenosis. In the past reported cases, including ours, a side-to-side anastomosis, an end-to-end anastomosis by layer-to-layer suture, or a functional end-to-end method was performed at the second operation, and recurrence was not observed. Anastomosis techniques other than the end-to-end Albert-Lembert method may contribute to prevent the occurrence of intussusception after FJT.

Some authors reported that the fixation of oral side of the jejunum adjacent to the anastomotic site may have increased the incidence of abnormal bowel motility resulting in intussusception [13,15]. Therefore, they recommended that the free jejunal graft should be harvested at a site removed from the Treitz ligament to avoid postoperative intussusception [15]. Although the implication of the distance from the Treitz ligament and the influence of the oral side fixation adjacent to the anastomosis on the occurrence of intussusception have not been fully understood, we may have to care about the distance from the Treitz ligament to the anastomosis after harvest of a free jejunal graft.

## Conclusions

In summary, we experienced two cases of intussusception causing a postoperative intestinal obstruction following FJT. In order to prevent the occurrence of postoperative intussusception, we recommend to avoid reconstruction by Albert-Lembert end-to-end anastomosis and to harvest a free jejunal graft as far from the Treitz ligament as possible.

## Consent

Written informed consent was obtained from the patient for publication of this case report and any accompanying images. A copy of the written consent is available for review by the Editor-in-Chief of this journal.

## Abbreviations

FJT: free jejunum transfer
POD: on postoperative day
CT: computed tomography
